# Identification and characterization of naturally occurring splice variants of SAMHD1

**DOI:** 10.1186/1742-4690-9-86

**Published:** 2012-10-23

**Authors:** Sarah Welbourn, Eri Miyagi, Tommy E White, Felipe Diaz-Griffero, Klaus Strebel

**Affiliations:** 1 Viral Biochemistry Section, Laboratory of Molecular Microbiology, National Institute of Allergy and Infectious Diseases, NIH, Building 4, Room 310; 4 Center Drive, MSC 0460, Bethesda, MD, 20892-0460, USA; 2 Department of Microbiology and Immunology, Albert Einstein College of Medicine Bronx, New York, NY, 10461, USA

**Keywords:** Vpx, SAMHD1, Splicing, Gene regulation

## Abstract

**Background:**

Sterile Alpha Motif and HD domain-containing protein 1 (SAMHD1) is a recently identified host factor that restricts HIV-1 replication in dendritic and myeloid cells. SAMHD1 is a dNTPase that presumably reduces the cellular dNTP levels to levels too low for retroviral reverse transcription to occur. However, HIV-2 and SIV encoded Vpx counteracts the antiviral effects of SAMHD1 by targeting the protein for proteasomal degradation. SAMHD1 is encoded by a multiply spliced mRNA and consists of 16 coding exons.

**Results:**

Here, we identified two naturally occurring splice variants lacking exons 8–9 and 14, respectively. Like wildtype SAMHD1, both splice variants localize primarily to the nucleus, interact with Vpx, and retain some sensitivity to Vpx-dependent degradation. However, the splice variants differ from full-length SAMHD1 in their metabolic stability and catalytic activity. While full-length SAMHD1 is metabolically stable in uninfected cells, both splice variants were inherently metabolically unstable and were rapidly degraded even in the absence of Vpx. Vpx strongly increased the rate of degradation of full-length SAMHD1 and further accelerated the degradation of the splice variants. However, the effect of Vpx on the splice variants was more modest due to the inherent instability of these proteins. Analysis of dNTPase activity indicates that neither splice variant is catalytically active.

**Conclusions:**

The identification of SAMHD1 splice variants exposes a potential regulatory mechanism that could enable the cell to control its dNTPase activity on a post-transcriptional level.

## Background

HIV-2 and many SIV isolates encode an accessory protein, Vpx, that is required for replication in myeloid cells [1-4]. HIV-1 does not encode a *vpx* gene; interestingly, however, the presence of Vpx enhances replication of HIV-1 in monocyte-derived macrophages, dendritic cells, and the differentiated THP-1 cell line [1,5-8] suggesting the presence of a Vpx-sensitive host restriction factor. Also, Vpx was shown to rescue HIV-1 but not HIV-2 or SIV from an interferon-induced antiviral state [9]. A Vpx-sensitive restriction factor was recently identified as Sterile Alpha Motif and HD domain-containing protein 1 (SAMHD1), which was found to be targeted for proteasomal degradation by Vpx [10-13]. Mutations in the SAMHD1 gene have been implicated with Aicardi-Goutieres Syndrome (AGS), a disease that is associated with increased production of interferon-alpha and thus mimics congenital virus infections [14,15]. This suggests SAMHD1, along with other AGS-associated proteins (e.g. TREX1 and RNASEH2), may be involved in the regulation of the innate immune response [16]. Moreover, SAMHD1 was shown to contribute to the restriction of HIV-1 in resting CD4+ T cells [17,18]. SAMHD1 has recently been shown to possess dGTP-dependent dNTPase activity [19,20], and the current hypothesis is that SAMHD1 is able to deplete the intracellular pool of dNTPs in susceptible cell types to levels below that required for reverse transcription, thus resulting in restriction of HIV-1 replication [19-22].

Here, we describe the identification and characterization of two SAMHD1 splice variants that are expressed naturally together with full-length SAMHD1 in a variety of cell types. The splice variants identified either lack exons 8–9 (Δ8-9), eliminating a C-terminal portion of the HD domain, or exon 14 (Δ14). Like wildtype SAMHD1, the Δ8-9 and Δ14 splice variants exhibit nuclear localization, and they interact with Vpx. However, unlike wildtype SAMHD1, which is stable in the absence of Vpx, both splice variants are inherently unstable and are rapidly degraded even in the absence of Vpx. Pulse/chase analyses confirmed that Vpx had a strong impact on the degradation of full-length SAMHD1. Addition of Vpx also increased the turnover of the splice variants. However, this effect was more modest because of the inherent instability of these proteins in the absence of Vpx. Neither splice variant exhibited dNTPase activity in an *in vitro* assay, suggesting they also lack antiviral activity. The identification of SAMHD1 splice variants exposes a potential regulatory mechanism that could enable a cell to control its dNTPase activity at a post-transcriptional level.

## Results

### Cloning and expression of SAMHD1 splice variants

**Figure 1:**
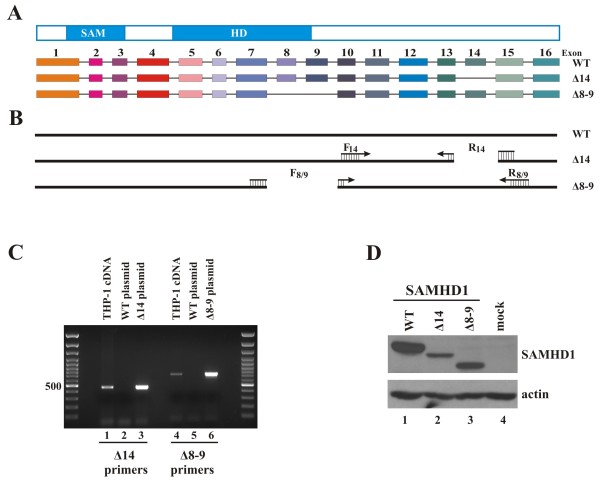
**Identification and characterization of SAMHD1 splice variants.** (**A**) Schematic representation of the SAMHD1 protein and the SAMHD1 splice variants cloned from PMA-treated THP-1 cells. (**B**) Location of the primers used for the specific amplification of SAMHD1 splice variants. Δ14 primers consist of a forward primer (F14) located in exon 10 and a reverse primer (R14) spanning the junction of exons 13 and 15. The resulting PCR product is predicted to be 462 bp long. Δ8-9 primers consist of a forward primer (F8/9) spanning the junction of exons 7 and 10 and a reverse primer R8/9 in exon 15, resulting in a predicted PCR product of 711 bp. (**C**) cDNA pools were generated from THP-1 cells treated with 100 nM PMA for 24 h and used as template for a PCR reaction using Taq polymerase and the primer sets described in panel B. Plasmid controls consisted of 1 ng of pcDNA-SAMHD1 WT, Δ14, or Δ8-9 used as template in the PCR reaction, as indicated. (**D**) HeLa cells were transfected with 5 μg each of pcDNA-SAMHD1 WT, Δ14, or Δ8-9 as indicated. Total cell extracts were prepared 24 h later and separated on 10% SDS-PAGE. Immunoblot analysis was performed with rabbit polyclonal antibodies specific to SAMHD1 (SAM416) and actin, as indicated.

To characterize SAMHD1 and its role in restricting lentiviral replication, we cloned SAMHD1 from the mRNA pool of PMA-differentiated THP-1 cells. SAMHD1 specific cDNA was cloned into pcDNA3.1 either in untagged form or with an N-terminal HA tag. Interestingly, in addition to the complete 16-exon gene encoding the 626 residue full-length SAMHD1 protein, we identified two splice variants lacking exons 8 plus 9 (corresponding to residues 285–354) or exon 14 (residues 502–536), respectively (Figure 1A). To confirm the presence of these splice variants in differentiated THP-1 cells, splice-junction specific primers were designed for the selective amplification of alternatively spliced mRNA (Figure 1B). SAMHD1 WT, Δ14, and Δ8-9 plasmid DNAs were used as specificity controls. As expected, the primer set F_14_/R_14_ specific for SAMHD1Δ14 showed a PCR product with SAMHD1Δ14 DNA as template but not when the wildtype plasmid was used (Figure 1C, lanes 2 and 3). The Δ8-9 primer set F_8/9_/R_8/9_ showed similar specificity for the Δ8-9 construct (Figure 1C, lanes 5 and 6). Importantly, when cDNA from PMA-differentiated THP-1 cells was used as a template, both primer sets amplified a band of appropriate size indicating the splice variants are genuinely present in the mRNA pool of these cells (Figure 1C, lanes 1 and 4). Similar results were observed for SAMHD1 mRNA from 293 T cells and primary CD4+ T lymphocytes (data not shown). Notably, HeLa cells express very low amounts of SAMHD1; and, consistent with this, splice variants were not identified in these cells (data not shown). Thus, expression of SAMHD1 splice variants is not a unique property of THP-1 cells but was found in other SAMHD1-positive cell lines and primary human T cells tested.

**Figure 2:**
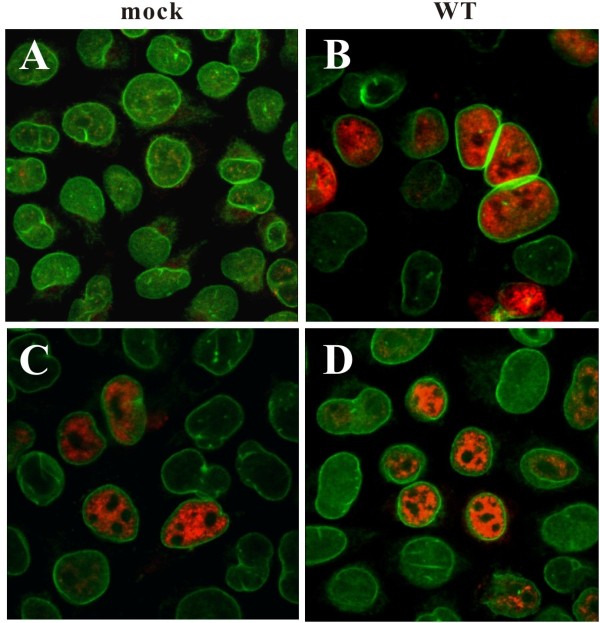
**Immunofluorescence analysis of SAMHD1 WT and splice variants.** HeLa cells were either mock transfected (panel **A**) or transfected with 1 μg of pcDNA-SAMHD1 WT (panel **B**), 5 μg of Δ14 (panel **C**), or 4 μg of Δ8-9 (panel **D**). Total amounts of transfected DNA were adjusted to 5 μg with empty vector DNA as necessary. After the transfection, cells were replated onto cover slips, grown for 24 h, and then fixed with methanol (10 min, -20°C). Cells were dually stained with rabbit polyclonal antibody to SAMHD1 (SAM416; red) and a mouse monoclonal antibody to lamin-B (green). Samples were analyzed on a confocal microscope as detailed in Methods.

Differential splicing might be an important mechanism for the cell to regulate SAMHD1 and its functions. Therefore, we set out to study the functional properties of the splice variants as compared to the wildtype protein. For this purpose, we generated a polyclonal antibody to SAMHD1 (SAM416) using recombinant protein purified from *E. coli* as previously described [23]. The antibody readily identified wildtype SAMHD1 and its splice variants by immunoblotting (Figure 1D) and immunocytochemistry (see Figure 2). Interestingly, transfection of constant levels of plasmid DNAs yielded significant differences in the expression level of the splice variants relative to the full-length protein. In particular, SAMHD1Δ14 consistently yielded a lower signal than the wildtype protein in immunoblot analyses (Figure 1D, lane 2). This was unlikely due to differential recognition by SAM416 since N-terminally HA-tagged constructs detected with an anti-HA antibody showed similar differences in signal intensities (data not shown). 

### SAMHD1 splice variants exhibit the same nuclear localization as the wildtype protein

While wildtype SAMHD1 localizes almost exclusively to the nucleus [14], mutants associated with AGS cause the protein to be at least partially localized to the cytoplasm [24], suggesting a possible correlation between SAMHD1 nuclear localization and function. However, recent evidence has suggested that while SAMHD1 nuclear localization may not be strictly required for HIV-1 restriction, it may be important for the initiation of Vpx-dependent degradation of SAMHD1 [25]. Immunocytochemistry of untagged wildtype SAMHD1 transfected into HeLa cells confirmed the nuclear localization of SAMHD1 (Figure 2, panel B). Similarly, SAMHD1Δ14 and Δ8-9 revealed exclusively nuclear localization in the vast majority of transfected cells (Figure 2, panels C and D). This indicates that sequences encoded by exons 8–9 and 14 are not critical for determining the subcellular distribution of SAMHD1. Indeed, a KRPR nuclear localization sequence has recently been mapped to the N-terminus of SAMHD1 [25].

### SAMHD1 splice variants interact with Vpx

**Figure 3:**
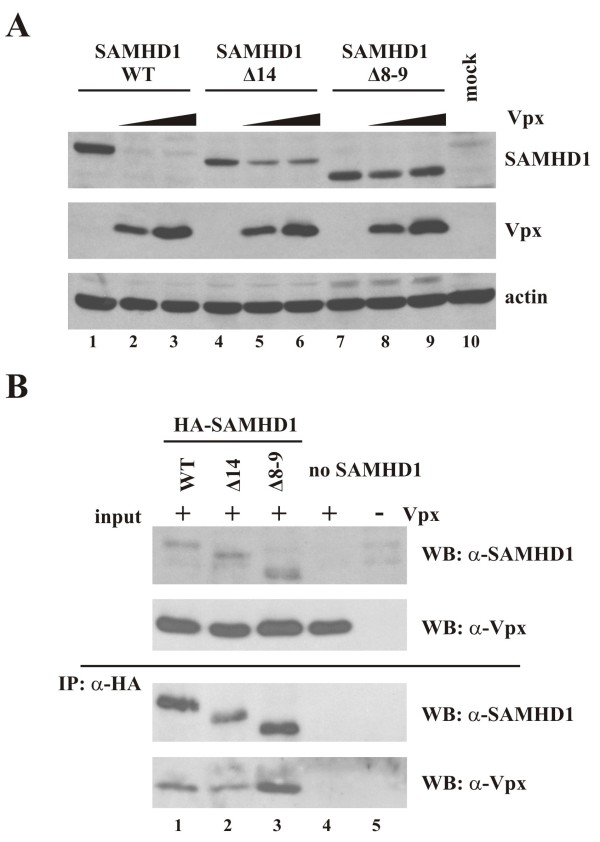
**Interaction of Vpx and SAMHD1 splice variants.** (**A**) HeLa cells were transfected with pcDNA-SAMHD1 WT (1 μg), Δ14 (5 μg), or Δ8-9 (4 μg) as indicated, either without Vpx (lanes 1, 4, 7) or with increasing amounts (0, 0.25, 0.5 μg) of pCMV-Vpx_mac239_. Whole cell extracts were prepared 24 h after transfection and separated on 10% (SAMHD1, actin) or 15% (Vpx) SDS-PAGE. Immunoblot analysis was performed with rabbit polyclonal antibodies specific to SAMHD1 and SIV_mac239_ Vpx. The SAMHD1 blot was subsequently reprobed with a rabbit polyclonal antibody to actin. (**B**) Vpx interacts with SAMHD1 splice variants. HeLa cells were transfected with pcDNA-HA-SAMHD1 WT (2 μg), Δ14 (5 μg), or Δ8-9 (3 μg) together with 0.5 μg of pCMV-Vpx_mac239_. Total amounts of transfected DNA were adjusted to 5.5 μg with empty vector DNA as appropriate. MG132 (10 μM final concentration) was added to the samples 20 h post-transfection and cells were lysed 4 h later and processed for immunoprecipitation with HA-beads as described in Methods. Immunoprecipitated proteins (IP: α-HA) together with input lysate (input) were separated by SDS-PAGE and immunoblot analysis was performed with antibodies to SAMHD1 or Vpx as indicated.

Vpx has been shown to render monocytic cells permissive to HIV-1 infection by binding to and causing the proteosomal degradation of SAMHD1 [10,11]. In order to determine whether SAMHD1Δ14 and Δ8-9 are subject to Vpx-dependent degradation, SAMHD1 WT, Δ14, and Δ8-9 were transfected into HeLa cells together with increasing amounts of Vpx from SIV_mac239_. To adjust for the lower expression of the splice variants, amounts of transfected DNA were modified to achieve comparable expression of wildtype SAMHD1 and the splice variants in the absence of Vpx (Figure 3A, lanes 1, 4, and 7). Immunoblot analysis showed that levels of wildtype SAMHD1 were effectively reduced in Vpx expressing cells (Figure 3A, compare lanes 1 & 2). In contrast, Vpx had a more subtle effect on the steady-state expression of SAMHD1 splice variants (Figure 3A, compare lanes 4 and 5, and 7 and 8). In particular, SAMHD1Δ8-9 steady-state expression appeared to be much less affected by Vpx than the wildtype protein (Figure 3A, compare lanes 1 and 2 with lanes 7 and 8). Interestingly, the effects of Vpx on SAMHD1 levels did not increase at higher levels of Vpx (Figure 3A, compare lanes 2 and 3, 5 and 6, 8 and 9) suggesting that Vpx is saturating in our system even at the lowest level tested. 

To investigate whether the less pronounced effect of Vpx on the expression of SAMHD1 splice variants was due to a lack of protein-protein interaction, co-immunoprecipitation studies were performed employing N-terminally HA-tagged SAMHD1 variants, which were cotransfected into HeLa cells together with Vpx (Figure 3B). To minimize effects due to SAMHD1 degradation, cells were treated with the proteasome inhibitor MG132 for 4 h prior to cell lysis. Consistent with previous reports [10-12], wildtype SAMHD1 interacted with Vpx as indicated by the co-immunoprecipitation of Vpx in the SAMHD1 pulldowns (Figure 3B, bottom panel, lane 1). Interestingly, both SAMHD1 splice variants also interacted with Vpx as indicated by the efficient co-immunoprecipitation of Vpx (Figure 3B, lanes 2–3). Vpx was not pulled down by HA antibodies in the absence of SAMHD1 (Figure 3B, lane 4) attesting to the specificity of the assay. These results indicate that regions in SAMHD1 missing in the splice variants are not strictly required for Vpx binding. This is consistent with recent data suggesting a C-terminal region of SAMHD1 is involved in the interaction with Vpx [26,27].

### SAMHD1 splice variants remain sensitive to Vpx-induced degradation

**Figure 4:**
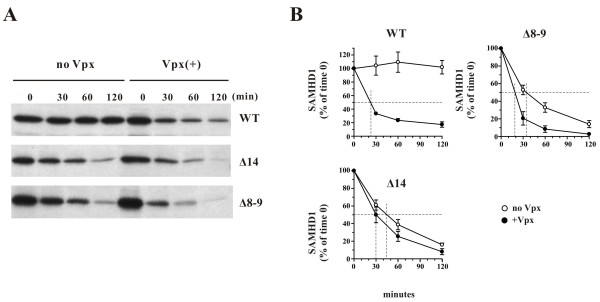
**Pulse-chase analysis of SAMHD1 in the presence or absence of Vpx.** HeLa cells were transfected with 4.5 μg each of pcDNA-SAMHD1 WT, Δ14, or Δ8-9 either with 0.5 μg of empty vector DNA (no Vpx) or together with 0.5 μg of pCMV-Vpx_mac239_ (Vpx(+)). Thus, the SAMHD1:Vpx DNA ratio was 10:1 for all samples. Pulse-chase analysis was performed 24 h later by labeling cells for 15 minutes with [^35^S]-methionine/cysteine and chasing for up to 2 h as described in Methods. Labeled SAMHD1 proteins were immunoprecipitated using SAM416 antiserum, separated on SDS-PAGE, and visualized by fluorography. Protein bands were quantified by PhosphorImage analysis and plotted as a function of time. Signal intensity at the 0 time points was defined as 100%. Error bars reflect the standard error of the mean from two independent experiments.

To assess whether the differences in SAMHD1 levels observed for wildtype SAMHD1 and its splice variants in Figure 3A in the presence of Vpx are due to differential sensitivity to Vpx-dependent degradation, pulse-chase analyses were performed and proteins were immunoprecipitated with SAM416 antibody as detailed in Methods (Figure 4A). Note that in this experiment equal amounts of SAMHD1 vectors were transfected. Protein bands were quantified by PhosphorImage analysis (Figure 4B). We found that wildtype SAMHD1 was metabolically stable in the absence of Vpx with no apparent loss of protein during the 2 h observation period (Figure 4B WT, open circles). However, in the presence of Vpx, SAMHD1 was rapidly degraded with a half-life of ~25 minutes (Figure 4B WT, closed circles). Interestingly, at the pulse time point, the signals obtained for the splice variants were comparable to those of wildtype SAMHD1 (Figure 4A, lanes “0”). This suggests that differences in steady state levels observed in Figure 3A are not due to differences in mRNA stability or protein expression. Instead, both the Δ14 and Δ8-9 splice variants were inherently unstable even in the absence of Vpx and had half-lives of ~45 and ~35 min, respectively (Figure 4B Δ8-9 & Δ14, open circles). Co-expression of Vpx further increased their rate of degradation (Figure 4B, Δ8-9 & Δ14, closed circles). Because of the inherent instability of the SAMHD1 splice variants, the differences in degradation kinetics in the presence or absence of Vpx were less pronounced than in the wildtype protein. However, the half-lives of ~20-30 minutes for the splice variants, which result from the combined effects of their instability plus the additional impact of Vpx, are very similar to the 25 minutes half-life observed for wildtype SAMHD1 in the presence of Vpx.

### SAMHD1 splice variants lack dNTPase activity

**Figure 5:**
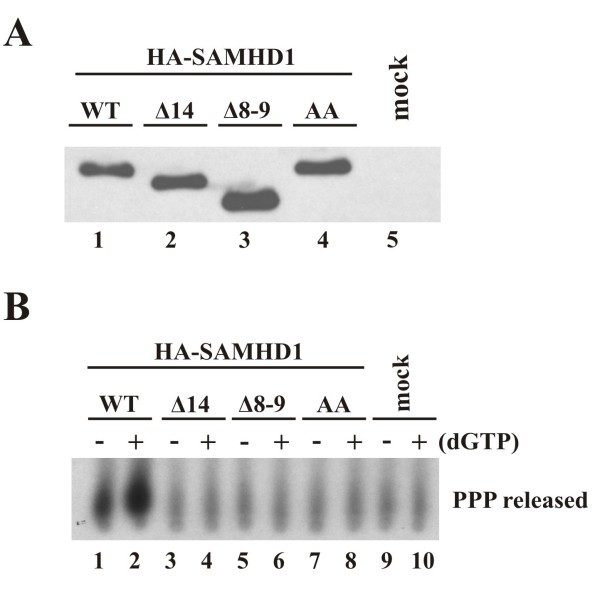
**Analysis of dNTPase activity in SAMHD1 splice variants.** (**A**) HeLa cells were transfected with pcDNA-HA-SAMHD1 WT, Δ14, Δ8-9 or a catalytically inactive SAMHD1 mutant, H206A/D207A (AA). Amounts of transfected DNA were adjusted to achieve comparable protein levels. Cell extracts were prepared 24 h later and used for immunoprecipitation with HA-beads as described in Methods. 15% of the total immunoprecipitated SAMHD1 proteins were separated on SDS-PAGE and detected by immunoblot using SAMHD1 specific antiserum. (**B**) The remaining immunoprecipitated protein was used in a dTTPase assay directly on the beads using 0.5 μCi α-^32^P] dTTP and conditions reported by Lahouassa *et al.*[21] and described in Methods. Where indicated, 200 μM dGTP was added to the reaction buffer. Released triphosphate (PPP) was separated by polyethyleneimine (PEI)-cellulose thin layer chromatography using 0.8 M LiCl as the mobile phase.

Several studies have recently proposed that the HIV-1 restrictive activity of SAMHD1 is due to its ability to cleave dNTPs [19-21]. This activity is thought to reduce the dNTP pool to a level insufficient to support reverse transcription of the retroviral genome, as also suggested by Kim *et al.* 2012 [22]. Based on these findings, we next determined the dNTPase activity of our SAMHD1 splice variants. In order to do so, HA-SAMHD1 proteins were immunoprecipitated from transfected HeLa cells using HA beads. As before, experimental parameters were adjusted to yield comparable levels of protein to use in the assay (Figure 5A, lanes 1–3). As a control, a catalytically inactive mutant, SAMHD1 AA [19], was constructed as detailed in Methods and included in this analysis (Figure 5A, lane 4). Immunoprecipitates were used directly in a dTTPase assay as detailed in Methods using the conditions described by Lahouassa *et al.*[21]. In our assay, SAMHD1 dNTPase activity results in the release of triphosphate (PP^32^P) from α-^32^P]-dTTP, which was separated from uncleaved dTTP by thin layer chromatography. As expected, wildtype SAMHD1 exhibited catalytic activity (Figure 5B, lane 1), and this activity was enhanced in the presence of unlabeled dGTP (Figure 5B, lane 2). The catalytically inactive SAMHD1 AA variant failed to release more than background levels of triphosphate from the dTTP substrate even when dGTP was added (Figure 5B, compare lanes 7–8 and 9–10). Of note, neither SAMHD1 Δ14 (Figure 5B, lanes 3–4) nor Δ8-9 (lanes 5–6) was able to release triphosphate in this assay indicating that these splice variants are catalytically inactive. 

To assess the ability of our splice variants to restrict HIV-1 infection, we stably transduced U937 cells using a lentiviral vector system containing the indicated splicing variants ( Additional file 1: Figure S1A). Wild type SAMHD1 clearly restricted HIV-1 infection of U937 cells ( Additional file 1: Figure S1B). In contrast, neither one of the two splice variants inhibited HIV-1 infection ( Additional file 1: Figure S1B). Of note, expression of the splice variants was poor when compared to the wild type protein ( Additional file 1: Figure S1A). When wildtype SAMHD1 expression was reduced to levels comparable to those observed for the splice variants by titrating the transducing vector, no restriction of HIV-1 was observed even with wild type SAMHD1 (data not shown). Therefore, the results from this experiment do not conclusively prove or disprove restriction capacity of the SAMHD1 splice variants. However, since dNTPase activity of SAMHD1 appears to be required for its antiviral activity [21], our results would suggest that the Δ14 and Δ8-9 splice variants lack restricting activity.

## Discussion

Nearly 90% of human gene sequences consist of introns that must be removed through splicing (for review see [28]). Alternative splicing allows the production of different proteins from a single gene. Indeed, about 80% of genetic variability introduced through alternative splicing falls within open reading frames [29]. Recent genome-wide analyses of alternative splicing indicate that up to 70% of human genes may have alternative splice forms, suggesting that alternative splicing together with various posttranslational modifications plays a major role in expanding proteome complexity [30]. Proteins produced by alternative splicing can differ from the full-length product in their biophysical and biological properties at the level of protein stability, protein modification, intracellular localization, or surface expression [28]. Thus, alternative splicing is a fundamental aspect of post-transcriptional gene regulation in eukaryotic cells with significant functional implications.

The 3.2 kb SAMHD1 mRNA (NM_015474) consists of 16 exons that have to be post-transcriptionally spliced from the 62 kb pre-mRNA. Recently, a G to A mutation in the SAMHD1 gene was found in patients with cerebral vasculopathy [31]. The mutation localized to the exon 12 splice-acceptor site and resulted in the skipping of exon 13. Cell lines carrying this defect in SAMHD1 failed to show SAMHD1 expression suggesting that the exon 13-deficient splice variant was highly unstable [31]. While the splicing defect observed in cerebral vasculopathy patients was not caused by alternative splicing but, instead, was induced by a genetic mutation, it nevertheless exemplifies the dramatic effects variation in splicing can have on the biophysical and biomedical properties of the resulting protein.

**Figure 6:**
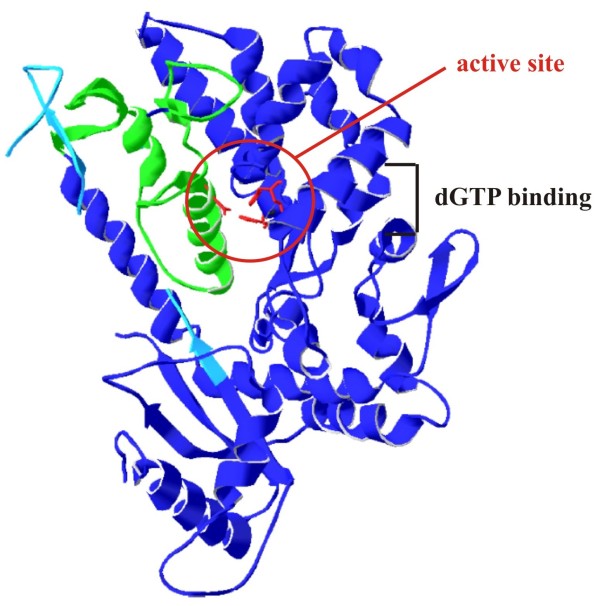
**Location of SAMHD1 exons 8–9 and exon 14 sequences on SAMHD1 structural model.** A representation of the SAMHD1 crystal structure was generated using Swiss PDB viewer ( [32] and http://www.expasy.org/spdbv/) based on published coordinates from Goldstone *et al.* (PDB ID: 3U1N [19]). Active site residues His_167_, His_206_, Asp_207_ and Asp_311_ are shown in red and are highlighted by a red circle. The region encoded by exons 8 and 9 (residues 285–354) is shown in green. The region encoded by exon 14 (residues 502–536), for which only partial structural information is available, is shown in light blue. The dGTP binding region of SAMHD1 as determined by Goldstone *et al.*[19] is indicated as well

In contrast to the exon 13 skipping associated with cerebral vasculopathy, the splice variants identified in our study are caused by alternative splicing. Consequently, the proteins expressed from the alternatively spliced mRNAs would be co-expressed together with full-length SAMHD1 protein. Interestingly, the production of these splice variants is not limited to THP-1 cells. In fact, both the Δ8-9 and Δ14 variants were identified in other SAMHD1-expressing cells analyzed, including primary human CD4^+^ T cells and the 293 T cell line (data not shown). This suggests that production of SAMHD1 splice variants is a general property of SAMHD1 gene expression and thus may have functional significance. The regions missing in the splice variants identified in this study are visualized in Figure 6 on a representation of the published crystal structure of SAMHD1 [19]. Active site residues are depicted in red. The region that is encoded by exons 8 and 9 is shown in green and encompasses alpha helix 11, which contributes Asp_311_ to the active site. As mutation of this residue to alanine has been shown to render SAMHD1 catalytically inactive [19], it is not surprising that SAMHD1 Δ8-9, in which this whole region is missing is inactive. Why the Δ14 splice variant is catalytically inactive is less obvious. Exon 14 is part of the C-terminal domain of SAMHD1 [19], distinct from the active site, and there is only partial structural information available for this region (Figure 6, light blue). It is possible that part of this region lies on top of the active site cavity and may be required for SAMHD1 function. However, it is also possible that removal of exon 14 sufficiently changes or destabilizes the structure to impact the active site and/or the more distal allosteric dGTP binding region of SAMHD1. 

The fact that our SAMHD1 splice variants are catalytically inactive but retain the ability to bind Vpx, raises the possibility that these proteins might act as decoys for full-length SAMHD1 and interfere with the ability of Vpx to target the functional protein. Interference studies are currently in progress to experimentally test this possibility. However, given the inherent instability of the two splice variants identified in our study, it seems more plausible to speculate that alternative splicing of the SAMHD1 pre-mRNA is a regulatory mechanism employed by the cells to modulate expression and function of catalytically active SAMHD1 at a post-transcriptional level. This view is supported by our finding that both splice variants can be identified in multiple cell types. Because of its inherent dNTPase activity, SAMHD1 is likely to be involved in regulating the cellular dNTP levels. In non-dividing cells, such as terminally differentiated macrophages or dendritic cells, there is no active host DNA replication and SAMHD1 activity contributes to keeping the cellular dNTP level low. This might in fact explain, at least in part, the resistance of non-dividing cells to infection by HIV-1 [21,22,33]. In contrast, in actively dividing cells, e.g. immortalized cell lines such as 293 T or HeLa or in undifferentiated THP-1 cells, expression of an active dNTPase might be counterproductive. Still, we noted that the levels of SAMHD1 in differentiated (non-dividing) and undifferentiated (dividing) THP-1 cells are indistinguishable (data not shown) suggesting that dNTP levels in these cells may be regulated by other yet to be identified mechanisms.

## Conclusions

While the SAMHD1 splice variants identified here are less stable than the full-length protein, it is possible that these or other yet to be identified forms of SAMHD1 may have functional roles in specific cell types or under specific conditions. In any case, characterization of different SAMHD1 splice variants, like those described here, not only yields important insight into potential functional differences of these proteins but also helps characterize the regions in SAMHD1 involved in dNTPase activity, HIV-1 restriction, and Vpx-dependent inactivation.

## Methods

### Cell culture and transfections

THP-1 cells were maintained in RPMI 1640 media containing 10% fetal bovine serum. For differentiation, 5 million cells were treated with 100 nM phorbol 12-myristate 13-acetate (PMA, Sigma-Aldrich, Inc., St. Louis MO) in a 6-well plate. Cells were washed after 24 h and left an additional 24 h in culture prior to harvest. HeLa cells were propagated in Dulbecco’s modified Eagles medium (DMEM) containing 10% fetal bovine serum. For transfection, HeLa cells were grown in 25 cm^2^ flasks to about 80% confluency (~3x 10^6^ cells). Cells were transfected using LipofectAMINE PLUS^TM^ (Invitrogen Corp, Carlsbad CA) following the manufacturer’s recommendations. A total of 5–6 μg of plasmid DNA per 25 cm^2^ flask was used. Where appropriate, empty vector DNA was used to adjust total DNA amounts. Samples were harvested 24 h post-transfection.

### Antibodies

Polyclonal antibodies to human SAMHD1 and SIV_mac239_ Vpx were generated in rabbits immunized with purified recombinant fusion proteins expressed in *E. coli*. A MS2-SAMHD1 fusion protein containing amino acids 1–91 of the MS2 replicase followed by approximately 100 amino acids of a SAMHD1 fragment beginning at amino acid 416 was used to generate the SAMHD1 antiserum (SAM416), whereas the MS2 replicase was fused to the full length Vpx protein for generation of Vpx antiserum. A polyclonal antibody to actin (Sigma-Aldrich, Inc., St. Louis MO; Cat# A-5060) was used as a loading control. A mouse monoclonal antibody to human lamin B (Accurate Chemical & Scientific Corp., Westbury NY; Cat# BMDV3002) was used to stain the nuclear lamina. Fluorescently conjugated secondary antibodies used for immunofluorescence were purchased from Jackson ImmunoResearch Laboratories Inc. (West Grove PA).

### Plasmids

cDNA was generated from total RNA from PMA-differentiated THP-1 cells using a Superscript III kit and an oligo dT primer (Invitrogen). The SAMHD1 specific sequence was then isolated by PCR from the cDNA pool using forward primer 5^′^ AAT AAG CTA GCG AAA CCA TGC AGC GAG CCG ATT CCG AG 3^′^ and reverse primer 5^′^ TGC TCT AGA TTA CAT TGG GTC ATC TTT AAA AAG CTG GAC 3^′^. The PCR primers include Bmt1 and XbaI restriction sites for insertion into the *Bmt*1/*Xba*1 site of pcDNA3.1-APOBEC3G-HA [34]. The reverse primer also contained a stop codon to prevent expression of the C-terminal vector HA tag. This strategy led to the generation of pcDNA-SAMHD1 WT encoding the full-length protein as well as pcDNA-SAMHD1Δ14 and pcDNA-SAMHD1Δ8-9, encoding splice variants lacking exons 14 and 8–9, respectively. N-terminally HA-tagged SAMHD1 constructs were generated from the untagged constructs using similar Bmt1/Xba1 primers with an N-terminal HA tag included in the forward primer (5^′^ AA CTA GCT AGC GAA ACC ATG TAT CCA TAT GAC GTT CCA GAT TAC GCT TGT ACA CAG CGA GCC GAT TCC GAG CAG 3^′^). An active site mutant (SAMHD1 AA) was created by introducing a H206A/D207A double alanine mutation into wildtype SAMHD1 using Quikchange mutagenesis (Stratagene, Agilent Technologies). Plasmid pCMV-Vpx_mac239_ was constructed by replacing the *Sal*I/*Not*I *vif* insert in pVif-HA [35] with the *vpx* gene from SIVmac239. For that purpose, *vpx* from SIV_mac239_ was PCR amplified using primers 5^′^ A CGC GTC GAC ACC ATG TCA GAT CCC AGG GAG AG 3^′^ (forward) and 5^′^ ATA GTT TAG CGG CCG CGG GTT ATG CTA GTC CTG GAG G 3^′^ (reverse). These primers include the Vpx start and stop codons and create SalI and NotI restriction sites for subcloning into pVif-HA. The resulting construct expresses untagged Vpx protein. In all cases, fidelity of DNA sequence and insertion of mutants was verified by sequencing.

### Immunoblotting

For immunoblot analysis of cell-associated proteins, whole cell lysates were prepared as follows: Cells were washed once with PBS, suspended in PBS (~200 μl/5x 10^6^ cells) and mixed with an equal volume of sample buffer (4% sodium dodecyl sulfate, 125 mM Tris–HCl, pH 6.8, 10% 2-mercaptoethanol, 10% glycerol, and 0.002% bromophenol blue). Proteins were solubilized by heating 10 to 15 minutes at 95°C with occasional vortexing. Cell lysates were subjected to SDS-PAGE; proteins were transferred to PVDF membranes and reacted with appropriate antibodies as described in the text. Membranes were then incubated with horseradish peroxidase-conjugated secondary antibodies (GE Healthcare, Piscataway NJ) and proteins were visualized by enhanced chemiluminescence (ECL, GE Healthcare, Piscataway NJ).

### Co-immunoprecipitation analysis

HeLa cells were transfected with expression vectors for HA-SAMHD1 and Vpx as indicated in the text. Cells were harvested 24 h post transfection, washed with cold PBS and lysed in lysis buffer A (50 mM Tris–HCl [pH 7.5], 150 mM NaCl, 1% [v/v] Triton X-100, 10% glycerol) at 4°C for 20 min, then clarified by centrifugation at 10,000 x g for 10 min at 4°C. Ten to fifteen percent of the lysate was used as an input control. The remaining lysate was used for immunoprecipitation of HA-tagged antigens. Cleared cell lysates were mixed with anti-HA antibody-conjugated agarose beads (Sigma-Aldrich, Inc., St. Louis MO) and incubated at 4°C for 4 h. Samples were then washed four times with lysis buffer A. Proteins were eluted by boiling beads in sample buffer and subjected to immunoblot analysis with antibodies to SAMHD1 and Vpx.

### Metabolic labeling and immunoprecipitation

For pulse/chase analyses, transfected cells were harvested by scraping, pelleting, and suspending in 5 ml labeling media lacking methionine and cysteine (MP Biomedicals, Solon OH). Cells were incubated for 15 minutes at 37°C to deplete the endogenous methionine and cysteine pool. Cells were then pelleted, suspended in 600 μl of labeling medium containing 350 μCi of Expres^35^S^35^S protein labeling mix (Perkin Elmer, Shelton CT) and pulse-labeled at 37°C for 15 minutes. Cells were pelleted, suspended in complete RPMI, and equal volumes were distributed into separate tubes (1 tube per time point) containing 1 ml each of pre-warmed complete RPMI. After indicated chase times, cells were pelleted, supernatants were discarded, and cell pellets were frozen on dry ice. Cells were lysed in 200 μl of lysis buffer B (50 mM Tris–HCl [pH 7.5], 150 mM NaCl, 1% [v/v] Triton X-100) and incubated on ice for 5 minutes. Cell lysates were pelleted at 13,000 × g for 2 minutes to remove insoluble material. Cleared lysates were incubated for 1 h at 4°C with antibody-conjugated protein A-Sepharose. Beads were washed twice with wash buffer (50 mM Tris pH 7.4, 300 mM NaCl, 0.1% Triton X-100). Bound proteins were eluted by heating in sample buffer for 10 min at 95°C, separated by SDS-PAGE, and visualized by fluorography.

### Immunofluorescence and confocal microscopy

HeLa cells were transfected as indicated in the text. Transfected cells were trypsinized 3 h later and single-cell suspensions were distributed into 12 well plates containing 0.13 mm cover slips. Cells were grown for overnight at 37°C in DMEM containing 10% FBS. Cells were fixed in 10% methanol for 10 min at −20°C. Cells were blocked with 1% BSA in PBS for 30 minutes. For antibody staining, coverslips were incubated with appropriate primary antibodies in 1% BSA in PBS for 45 minutes at 37°C. Cells were washed with PBS and incubated with appropriate secondary antibodies for 45 minutes at 37°C. Cells were then washed twice with PBS and mounted onto microscope slides with glycerol gelatin (Sigma-Aldrich Inc., St. Louis MO) containing 0.1 M N-propyl gallate (Sigma) to prevent photo bleaching. Samples were analyzed on a Zeiss LSM410 inverted laser scanning microscope equipped with a krypton/argon mixed-gas laser. Images were acquired with a Plan-Apochromat 63x/1.4 oil immersion objective (Zeiss).

### Analysis of the expression of SAMHD1 splice variants

cDNA pools were generated from THP-1 cells treated with 100 nM PMA for 24 h and used as template for a PCR reaction using Taq polymerase (Roche) and the following primer sets: Δ14 primers consist of a forward primer (F14) located in exon 10 (5^′^ GAA GTT GGA AAT CTG TAT GAC ATG TTC CAC 3^′^) and a reverse primer (R14) spanning the junction of exons 13 and 15 (5^′^ CTC TGG CAG AAG TTG TGA AAC ATC 3^′^). Δ8-9 primers consist of a forward primer (F8/9) spanning the junction of exons 7 and 10 (5^′^ CTT GAA TCA CCT GTC GAA GAT TCA TTG GAA G 3^′^) and a reverse primer R8/9 in exon 15 (5^′^ CTG CGG CTT GGT GAA ATT TCT GTC TG 3^′^). Plasmid controls consisted of 1 ng of pcDNA-SAMHD1 WT, Δ14, or Δ8-9 used as template in the PCR reaction.

### dNTPase assay

HA-SAMHD1 proteins were isolated from HeLa cells and used in a dNTPase assay using the conditions described in Lahouassa *et al*. [21]. In brief, HeLa cells transfected with HA-SAMHD1 constructs were lysed in 50 mM Tris–HCl pH 8.0, 150 mM NaCl, 1% Triton at 4°C for 20 minutes, then clarified by centrifugation at 10,000 x g for 10 minutes at 4°C. Cleared cell lysates were mixed with anti-HA antibody-conjugated agarose beads (Sigma-Aldrich, Inc., St. Louis MO) and incubated at 4°C for 1.5-2 h. Samples were then washed three times with 50 mM Tris–HCl pH 8.0, 150 mM NaCl, 0.1% Triton and once with assay buffer A (50 mM Tris–HCl pH 8.0, 50 mM KCl, 5 mM MgCl_2_, 0.1% Triton). An equal volume of assay buffer A was then added to the bead pellet, 15% of the bead slurry reserved as an input control and the rest was mixed in a 1:1 ratio with assay buffer B (50 mM Tris–HCl pH8.0, 50 mM KCl, 5 mM MgCl_2_, 0.1% Triton, 400 μM dTTP, 0.5 μCi α-^32^P]-dTTP, +/− 400 μM dGTP). Reactions were incubated at 37°C for 3 h with occasional mixing and stopped by heating to 70°C for 5 minutes. A portion of the reaction products was separated out on polyethyleneimine (PEI)-cellulose thin layer chromatography plates (Sigma) using 0.8 M LiCl as the mobile phase.

## Competing interests

The authors declare that they have no competing interests.

## Authors’ contributions

SW and KS contributed to the concept and design of the study. SW and EM contributed to data acquisition and analysis. TEW and FD-G performed the HIV-1 restriction assay. SW and KS wrote the manuscript. All authors have read and approved the final manuscript.

## Supplementary Material

Additional file 1: Figure S1 Antiviral activity of naturally occurring splice variants of SAMHD1. Human monocytic U937 cells were transduced using a lentiviral vector system (pCDH; Systems Biosciences, Mountain View CA) expressing wild-type (WT) or the indicated splicing variants of SAMHD1. (**A**) Stable cell lines were selected by culturing for 4 days in the presence of 400 ng/ml of puromycin. SAMHD1 expression was then analyzed by immunoblotting using anti-SAMHD1 antibodies. The blot was then reprobed with antibodies to GADPH, which served as a loading control. (**B**) Differentiated U937 cells expressing the indicated protein were challenged with increasing amounts of HIV-1-GFP. Infection was determined by measuring the percentage of GFP-positive cells using a flow cytometer. As control, U937 cells stably transduced with the empty vector pCDH were challenged with HIV-1-GFP. Infection experiments were performed three times and a representative result is shown. Click here for file
