# Supplementary figures and images for: Identification and characterization of naturally occurring splice variants of SAMHD1
Source: Retrovirology. 2012 Oct 23;9:86. doi: 10.1186/1742-4690-9-86 (PMC3503569; doi:10.1186/1742-4690-9-86)

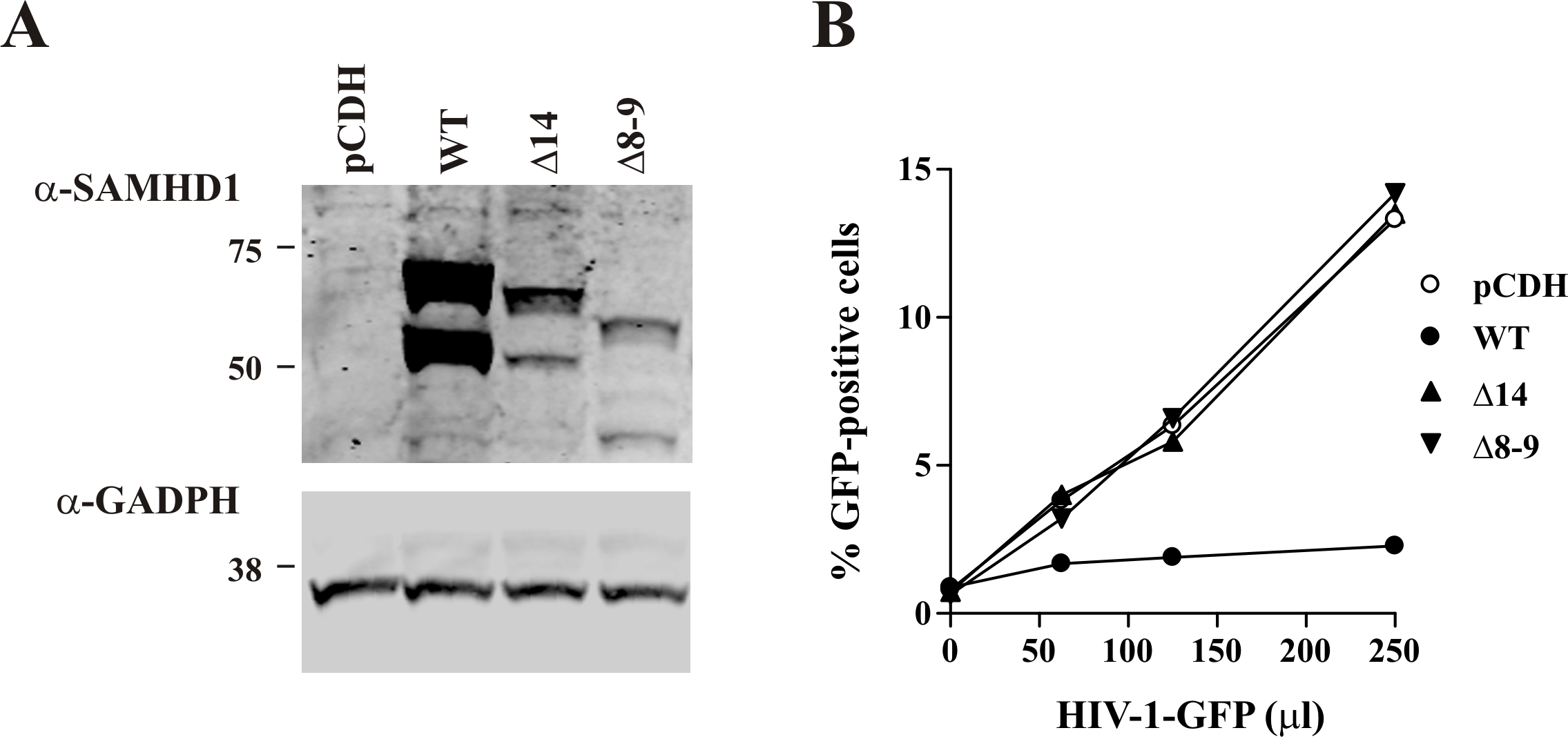

Supplement: Additional file 1: Figure S1 — Antiviral activity of naturally occurring splice variants of SAMHD1. Human monocytic U937 cells were transduced using a lentiviral vector system (pCDH; Systems Biosciences, Mountain View CA) expressing wild-type (WT) or the indicated splicing variants of SAMHD1. (A) Stable cell lines were selected by culturing for 4 days in the presence of 400 ng/ml of puromycin. SAMHD1 expression was then analyzed by immunoblotting using anti-SAMHD1 antibodies. The blot was then reprobed with antibodies to GADPH, which served as a loading control. (B) Differentiated U937 cells expressing the indicated protein were challenged with increasing amounts of HIV-1-GFP. Infection was determined by measuring the percentage of GFP-positive cells using a flow cytometer. As control, U937 cells stably transduced with the empty vector pCDH were challenged with HIV-1-GFP. Infection experiments were performed three times and a representative result is shown. [file 1742-4690-9-86-S1.tiff]
